# A retrospective study on titanium sensitivity: Patch test materials and manifestations

**DOI:** 10.1111/cod.13010

**Published:** 2018-05-24

**Authors:** Niels P. J. de Graaf, Albert J. Feilzer, Cees J. Kleverlaan, Hetty Bontkes, Sue Gibbs, Thomas Rustemeyer

**Affiliations:** ^1^ Department of Dermatology VU University Medical Centre Amsterdam The Netherlands; ^2^ Department of Oral Cell Biology, Academic Centre for Dentistry Amsterdam University of Amsterdam and Vrije Universiteit Amsterdam Amsterdam The Netherlands; ^3^ Department of Dental Materials Science, Academic Centre for Dentistry Amsterdam University of Amsterdam and Vrije Universiteit Amsterdam The Netherlands; ^4^ Unit Medical Immunology, Department of Clinical Chemistry VU University Medical Centre Amsterdam The Netherlands

**Keywords:** contact allergy, dermatitis, implants, medical device, patch test, titanium

## Abstract

**Background:**

Titanium is being increasingly used. Although it is considered to be a non‐allergenic material, allergic reactions to it have been reported. Titanium dioxide has been found to be an unreliable patch test material. Few studies to date have profiled titanium allergy, and it therefore remains difficult to distinguish its manifestations.

**Objectives:**

To evaluate alternatives for titanium dioxide as a patch test preparation, and to profile titanium reactions and manifestations.

**Methods:**

A retrospective chart review was conducted with 458 patients who underwent patch testing with at least 1 of 5 different titanium salts.

**Results:**

At least 1 positive result was noted in 5.7% of the patients. The frequency of positive results for the tested salts ranged from 0.9% to 7.9%. Titanium(IV) oxalate hydrate had the highest yield and titanium dioxide the lowest. Erythema, dermatitis and local swelling were the most common objective complaints. In 16 (61.5%) patients, the test result had partial or full clinical relevance.

**Conclusions:**

No titanium‐specific risk factors and clinical picture could be identified. Titanium dioxide is not adequately sensitive for identifying titanium allergy. The titanium salts seem to be possible superior patch test preparations, but appear to be unsuitable if used singly. The patient's medical history and clinical picture remain crucial in the diagnostic work‐up.

## 1 INTRODUCTION

Titanium (Ti) is a lustrous transition metal that is widely used as an implant material in medicine and dentistry, and, in its oxide form, as a white pigment in personal care products and food. It is often chosen as an implant material, owing to its corrosion resistance and good biocompatibility.^1^, ^2^ Many new Ti implant applications are being developed, and, because the age of the western population is increasing, human exposure to Ti is also increasing.^3^, ^4^

Although Ti is generally believed to be “hypo‐allergenic”, numerous articles have been published describing allergic reactions to Ti.^5^, ^6^, ^7^ The prevalence of Ti allergy is not known, but is estimated to be very low. Reports on Ti allergy have been summarized by Wood et al and Fage et al.^8^, ^9^ They describe adverse effects of Ti, namely, local and systemic symptoms such as local eczema in areas over an implant, pruritus, pain, chronic fatigue syndrome, and neurological symptoms.

Clinical experience with dental and orthopaedic implant patients suggests that Ti allergy occurs more often than patch tests indicate.^10^, ^11^, ^12^ The most widely used patch test preparation is Ti dioxide (TiO_2_), but it rarely confirms clinical suspicion. This might be explained by its poor solubility, resulting in inadequate skin penetration.^6^, ^7^ Other Ti salts, such as Ti(IV) chloride, Ti(II) sulfate, Ti(IV) diethanedioate, Ti salicylate, Ti(IV) tetrahydroxide, calcium titanate, Ti(III) nitride, and Ti(IV) oxalate hydrate, have been suggested, but only a few studies have actually examined the use of these salts.^9^, ^13^

In our clinic, during the past 10 years, different Ti test salts have been applied to evaluate possible sensitization to Ti. In this study, our objective was to report the frequency of positive patch test reactions to Ti dioxide and its alternatives applied in our clinic. In addition, this article describes the clinical presentation of Ti‐allergic patients in our clinic.

## 2 MATERIALS AND METHODS

After obtaining approval from our institutional review board, we performed a retrospective chart review on patients who underwent patch testing with ≥1 Ti test salts at the Allergy Unit of the Department of Dermatology at the VU University Medical Centre between January 1, 2004 and January 1, 2017. The Ti test salts that were used, the rationale for performing patch tests and the test results were recorded for all patients. For those with at least 1 positive patch test result, additional data were collected on sex, age, symptoms, implanted devices, clinical diagnoses, and relevance of the positive result.

All patients were tested with ≥1 Ti salts depending on the date of referral and the Ti salts used at that time (Table 1). The test materials used were TiO_2_, Ti(IV) oxalate hydrate, Ti(IV) isopropoxide, Ti lactate, and Ti citrate. Patch testing was performed with Van der Bend chambers (van der Bend, Brielle, The Netherlands). Patch test chambers were removed from the backs of the patients after 48 hours of exposure, and readings were performed on day (D) 2, D3, and D7. Positive reactions rated as +, ++ or +++ in accordance with the ICDRG/ESCD reading criteria were regarded as allergic,^14^ whereas doubtful reactions (?+) were not. The relevance of the positive reactions was assessed. An allergen was considered to be clinically relevant if: (1) the existence of exposure could be established, and (2) the patient's complaints could be explained (completely or partially) with regard to that exposure. The relevance was categorized as complete, partial, past, no and unknown relevance.^15^ Evaluations were performed by an experienced dermatologist.

**Table 1:** Amsterdam VU Medical Centre Department of Dermatology Allergy Unit titanium patch test salts

Allergen	Formula	Since year:	Concentration and vehicle	Manufacturer
Ti dioxide	TiO_2_	2004	“as is”	Material science laboratory ACTA
Ti(IV) isopropoxide	Ti[OCH(CH_3_)_2_]_4_	2009	10 ppm pet., 100 ppm pet., 500 ppm pet., 0.10% pet., 0.20% pet., 1% pet., 5% pet., 10% pet., 20% pet.	Material science laboratory ACTA
Ti(IV) oxalate hydrate	TiC_4_O_9_H_2_•*x*H_2_O	2012	5% pet.	Chemotechnique Diagnostics
Ti lactate	C_12_H_20_O_12_Ti	2014	0.04% pet., 0.08% pet., 0.16% pet., 0.24% pet.	SmartPractice Allergen Bank
Ti citrate	C_12_H_10_O_14_Ti_3_	2014	0.04% pet., 0.08% pet., 0.16% pet., 0.32% pet.	SmartPractice Allergen Bank

Abbreviation: ACTA, Academic Centre for Dentistry Amsterdam.

A 2‐tailed Fisher's exact test was used, as appropriate, to compare proportions of positive reactions to the Ti compounds in patients suspected of having Ti allergy with those in patients in the control group. The significance level for all analyses was *P* < .05.

## 3 RESULTS

A total of 458 patients were tested with ≥1 Ti salts (see Table S1 for combinations and numbers). At least 1 positive result was noted in 5.7% of patients (*n* = 26). The results of patch testing with the Ti salts are shown in Table 2. Most positive reactions were seen to Ti(IV) oxalate hydrate (7.9%; 216 tested). This was followed by Ti lactate (4.4%; 45 tested), Ti(IV) isopropoxide (2.9%, 272 tested), Ti citrate (2.2%; 45 tested), and TiO_2_ (0.9%; 329 tested). However, it should be noted that the patient groups for Ti citrate and Ti lactate were small as compared with those for the other salts.

**Table 2:** Patch test results per salt 2004 to 2016

		Positive	
	*N*	*n*	%
Tested for titanium sensitization	458	26	5.68
Titanium salt			
Ti(IV) oxalate hydrate	216	17	7.87
Ti(IV) isopropoxide	272	8	2.94
Ti citrate	45	1	2.22
Ti lactate	45	2	4.44
Ti dioxide	329	3	0.91

Patients could be divided into three groups: group 1 (*n* = 248) comprised patients suspected of having Ti allergy; group 2 (*n* = 163) comprised patients suspected of having a metal allergy other than to Ti; and group 3 (*n* = 47) comprised patients who were not exposed to Ti‐containing medical devices and did not have a specific history of Ti allergy, henceforth called the control group. In group 1, 22 patients showed positive test reactions (8.9%). In groups 2 and 3, 2 patients showed positive reactions (1.2% and 4.3%, respectively) (Table 3). The number of positive Ti reactions in patients suspected of having Ti allergy (group 1) was not statistically different from that in the control group (group 3) (*P* = .39). Exclusion of patients with positive patch test reactions to other metals did not influence the statistics.

**Table 3:** Patch test results per group and salt 2004 to 2016

		Positive	
	*N*	*n*	%
Group 1 (suspected titanium allergy)	248	22	8.87
Ti(IV) oxalate hydrate	174	15	8.62
Ti(IV) isopropoxide	224	8	3.57
100 ppm	224	1	0.44
1%	224	4	1.78
5%	224	1	0.46
10%	224	2	0.44
Ti citrate	37	1	2.70
0.16%	37	1	2.70
0.32%	37	1	2.70
Ti lactate	37	2	5.41
0.16%	37	2	5.41
Ti dioxide	139	1	0.72
Group 2 (suspected metal allergy)	163	2	1.23
Ti(IV) oxalate hydrate	4	0	0
Ti(IV) isopropoxide	4	0	0
Ti dioxide	159	2	1.26
Group 3 (control group)	47	2	4.26
Ti(IV) oxalate hydrate	38	2	5.26
Ti(IV) isopropoxide	44	0	0
Ti citrate	8	0	0
Ti lactate	8	0	0
Ti dioxide	31	0	0

Of the 26 positive patients, 23 reacted to only one Ti salt; the remaining 3 reacted to two Ti salts. Nickel and cobalt were the most frequent co‐reactants (both 19.2%). Notably, no Ti salt was found that was universally positive in patients who had positive test reactions. The mean age of the positive subjects was 55.2 years (range 20‐80 years); 53.8% were female. Most of the Ti‐positive patients had local symptoms such as pain, erythema, and dermatitis, but other symptoms, such as pruritus, impaired wound healing, and swelling, were also seen. Fifteen of the 26 (57.7%) had a proven Ti‐containing implant or reconstructive material. Most were orthopaedic and surgical (*n* = 10) or dental (*n* = 3), but 1 patient had a neurostimulator and another had an implanted insulin pump. In 16 of 26 (61.5%) positive patients, complete or partial clinical relevance of the positive result was determined. The demographic characteristics of the patients are summarized in Tables 4 and 5.

**Table 4:** Patch test results and characteristics of titanium‐positive patients

		Patch test							
Patient	Age (years)/sex	Ti dioxide	Ti oxalate hydrate	Ti isopropoxide	Ti citrate	Ti lactate	Clinical presentation	Ti implant(s)	Clinical relevance
1^a^	66/F	−	NT	−	−	+ (D3, D7)	Pr	Neurostimulator	Partial
2^a^	54/M	−	+ (D3)	−	−	−	ET, P, Pr	Orthopaedic	Complete
3^a^	74/F	NT	++ (D3)	−	NT	NT	S, De, P	Orthopaedic	Partial
4^a^	44/F	NT	+ (D3)	−	NT	NT	P, ES	−	Unknown
5^a^	47/M	−	+ (D3)	−	NT	NT	De	Insulin pump	Partial
6^a^	66/F	−	−	+ (D7)	NT	NT	PI	Dental	Complete
7^a^	80/M	NT	+ (D3)	−	NT	NT	BS	−	Unknown
8^a^	28/F	−	+ (D3, D7)	−	−	−	De	Orthopaedic	Partial
9^b^	64/F	−	+ (D3)	−	−	−	−	−	No
10^a^	27/M	+ (72 h)	NT	+ (D3)	NT	NT	DE	−	No
11^a^	57/F	NT	+ (D3)	−	NT	NT	S, ET, P	Orthopaedic	Past
12^a^	57/F	NT	+ (D2, D3)	−	NT	NT	Fa, P	−	No
13^b^	54/M	−	+ (D3, D7)	−	−	−	DE	−	No
14^a^	58/F	NT	+ (D7)	−	NT	NT	ET, S, P	−	Partial
15^c^	50/F	+ (72 h)	NT	NT	NT	NT	−	−	No
16^a^	75/M	−	−	+ (D3)	NT	NT	ET	Orthopaedic	Partial
17^a^	20/F	−	+ (D7)	−	NT	NT	Fa, IWH	Surgical	Partial
18^a^	58/F	NT	−	+ (D3)	NT	NT	De	Orthopaedic	Partial
19^a^	60/M	NT	−	+ (D3)	NT	NT	S, P	−	Unknown
20^a^	63/F	NT	+ (D3)	−	NT	NT	Pr	Orthopaedic	Partial
21^a^	64/M	NT	+ (D3)	+ (D3)	NT	NT	De, DE	Orthopaedic	Partial
22^c^	48/F	+ (72 h)	NT	NT	NT	NT	DE	−	Partial
23^a^	62/M	−	−	+ (D3)	−	+ (D3, D7)	De, DE	Orthopaedic	Partial
24^a^	40/M	−	+ (D3)	−	−	−	De	−	Unknown
25^a^	63/M	−	+ (D3)	−	+ (72 h)	−	ES	Dental	Partial
26^a^	57/M	NT	+ (D3)	+ (D3)	NT	NT	LE	Dental	Partial

Abbreviations: BS, burning sensation; De, dermatitis overlying the implant; DE, dermatitis elsewhere; ES, excessive saliva; ET, erythema; F, female; Fa, fatigue; IWH, impaired wound healing; LE, lichenoid eruption; M, male; NT, not tested; P, pain; PI, peri‐implantitis; Pr, pruritis; S, swelling.

a Group 1.

b Group 3.

c Group 2.

**Table 5:** Characteristics of 26 patients with a positive patch test reaction to titanium

Characteristic	Overall
Age (years)	
Mean	55.2
Range	20–74
Female, *n* (%)	14 (53.8)
Clinical relevance, *n* (%)	
No	5 (19.2)
Complete	2 (7.7)
Partial	14 (53.8)
Unknown	4 (15.4)
Past	1 (3.8)
Titanium implant	15 (57.7)
Dental	3 (20.0)
Orthopaedic/surgical	10 (66.7)
Other	2 (13.3)

## 4 DISCUSSION

We performed a retrospective study on all patients patch tested with Ti salts in our hospital. A key finding is that the frequency of Ti sensitivity in this large group of patients was 5.7%. This frequency was higher than the sensitivity found in a study by Sicilia et al, which was 0.6%, and that found in a study by Lhotka et al, which was 2%.^7^, ^16^ However, in these studies, only TiO_2_ was used, which might account for the difference in sensitization occurrence from that in our study. A study in Lithuania reported no positive patch test reaction to any of the 5 Ti salts present in their metal series.^17^ However, only a relatively small number of patients were tested. There are currently no reports in which a panel of Ti salts has been used on a large patch test population.

We tested a highly selected population; therefore, the high frequency of Ti sensitization that we found cannot be extrapolated to the general population. In the group of patients suspected of having Ti allergy, an even higher frequency of 8.9% was observed. Interestingly, this frequency was not statistically different from the frequency found in the control group (*P* = .39). This may be explained by the small sample size of the control group and the relatively low numbers of positive reactions within both groups. Also, the possible referral bias resulting from the selection of patients on the basis of their clinical history has to be taken into account. The potential differences in accuracy of the Ti salts should also be considered. The retrospective nature of this study makes it difficult to address these problems.

TiO_2_ is the most common patch test salt. It is an inert and highly insoluble material. Fage et al reviewed several studies on the penetration of TiO_2_, and did not find any studies that showed TiO_2_ penetration through the epidermis and into viable skin layers.^8^ Because of these characteristics, there is a high probability of false‐negative test results, regardless of the test concentration used. Nevertheless, in a summary of TiO_2_ patch testing reports, Wood et al described 21 patients with positive reactions.^9^ Unfortunately, no information on clinical relevance was provided. Our patch tests also showed some positive reactions to TiO_2_ (*n* = 3, 0.9%) in a large group of patients. In 1 patient (no. 22) with generalized eczema, TiO_2_ was tested because her eczema was exacerbated on the locations on which a Ti‐containing sunscreen was applied. The patient reacted positively to multiple possible components of sunscreens, eg, cosmetic preservatives, wool alcohol, fragrance mix, benzophenone, and Ti. Although partial relevance was identified, the role of Ti hypersensitivity can be considered to be insignificant in this case. No other reports of allergic reactions to Ti in sunscreens are known, and several studies have shown that TiO_2_ in sunscreens does not penetrate into the viable epidermis.^18^, ^19^ In 2 patients in group 2, positive reactions to TiO_2_ were found. In one patient (no. 10), the positive result was of no relevance for his dyshidrotic hand eczema, as no source of Ti contact could be found. The other patient (no. 15) with local dermatitis on a tattoo location had negative test results with all of the tattoo ink components. No source of Ti contact could be determined, and the result was therefore of no relevance. These results support the questionable relevance of positive TiO_2_ results. This is emphasized by the lack of studies in which a positive patch test result is supported by a positive in vitro test. This study confirms, in a large population, that TiO_2_ as a patch test preparation is of no value in clinical practice.

The use of Ti(IV) oxalate hydrate (TiC_4_O_9_H_2_•*x*H_2_O) as a patch test salt was first described in 1975.^20^ Only recently has it been as an alternative to TiO_2_ in clinical practice.^21^, ^22^ Chemotechnique Diagnostics initially labelled it as Ti(III) oxalate decahydrate; later, this was corrected to Ti(IV) oxalate hydrate. The test material itself was not changed. In our study, it was, notably, the highest‐scoring Ti salt, with positive reactions in 17 subjects (7.9%). Several cases have shown that Ti oxalate can show Ti sensitivity in TiO_2_‐negative patients.^23^, ^24^ This is similar to our experience, in which 8 patients who reacted positively to Ti oxalate were also tested with TiO_2_; none of the tests gave a positive result. However, the difference in frequency of positive Ti oxalate reactions between patients suspected of having Ti allergy and the control group was non‐significant (*P* = .74). As outlined above, this lack of significance may be attributable to unequal group sizes (*n* = 174 vs *n* = 38) and the retrospective nature of this study. This made statistical analysis for comparison of the groups difficult.

Nevertheless, positive reactions in the control group, in which sensitization to Ti is highly unlikely, highlight the possibility of false‐positive reactions. This is emphasized by the fact that 13 of the 17 positive subjects were tested with 2 Ti oxalate hydrate patches, but no concomitant reaction was seen in 8 (61.5%) of these subjects. Ti oxalate may be irritant in nature, owing to the low pH of 2.0‐3.0 when it is exposed to air. Arguably, this may not be the case when it is dissolved in petrolatum. A study by Bernard et al found only negative results in 30 control subjects who were patch tested with Ti oxalate, including in atopics.^23^ The authors therefore considered irritancy to be unlikely. To our knowledge, no large studies investigating the performance of Ti oxalate as a patch test salt exist. The present study shows that it is a superior salt for patch testing and can be of value in clinical practice. This is emphasized by the finding of complete or partial relevance in 73.3% of the Ti(IV) oxalate hydrate‐positive subjects in group 1. However, the significance of a positive reaction to Ti(IV) oxalate hydrate remains debatable, and has to be extensively examined on a case‐by‐case basis. Thus, results obtained with Ti oxalate should be interpreted with care until results from larger groups of tested patients are available for comparison.

Given the small test groups for Ti citrate and Ti lactate, no estimation can be made about their value in clinical practice. However, the good water solubility of Ti lactate and its ability to produce positive reactions makes it an interesting patch test salt for further investigation. This is emphasized by Basketter et al, who described 3 TiO_2_‐negative patients who had positive reactions to a complex Ti lactate.^25^ Investigating Ti isopropoxide could also be interesting, as it gave positive reactions in 8 patients. However, these results are enigmatic: even though Ti oxalate and Ti isopropoxide were simultaneously tested in 213 patients (including 20 of the 26 positive patients), there were only 2 concomitant reactions. From our data, no patient‐specific characteristics can be identified that could explain this. It may be interpreted as being attributable to false‐positive reactions to either of the materials. This interpretation is reinforced by the lack of multiple reactions to different Ti isopropoxide concentrations per patient, and the lack of reactions to higher concentrations in these patients. This might be the consequence of an inhomogeneous distribution of the allergen in the patch test vehicle. Therfore, no preferable concentration of Ti isopropoxide could be determined. Furthermore, no salt was capable of diagnosing all sensitized patients. Although it can be expected that testing with the expanded Ti series will enhance the detection of relevant positive reactions, the accuracy of these test salts can be questioned. Unfortunately, assessing the accuracy of each of these salts is not within the scope of this study. Additional prospective studies should evaluate the diagnostic accuracy, taking into account the lack of a gold reference standard.

Alternative diagnostic tools such as in vitro blood tests (lymphocyte transformation test [LTT] and memory lymphocyte immunostimulation assay) are available but have not yet been fully accepted as comparable alternative diagnostic tests. In our experience, the LTT is more appropriate for diagnosing sensitization in people who are sensitized and currently exposed, and less appropriate for people who are sensitized and not currently exposed. This topic needs more study. Therefore, from this study we conclude that, although the delayed positive reactions (mainly on D3) are in favour of a true allergic reaction, it cannot be confirmed whether Ti is a true sensitizer, whether the recorded positive results underestimate sensitization, or how the specific symptoms of a Ti sensitization are expressed. If Ti is a sensitizer, it is probably a weak one, and the fact that the majority of our patients showed mostly + reactions supports this notion. However, the test concentration, salt preparation and choice of vehicle may still need optimization. Studies on the immunological effects of Ti exposure have not yet led to a consensus on its sensitizing capabilities. A study by Lalor et al showed T lymphocytes and macrophages in the absence of B lymphocytes in the tissue of total hip revision surgery patients, suggesting Ti allergy.^10^ In contrast, Park et al,^26^ using a local lymph node assay, found that TiO_2_ was not a dermal sensitizer.

In both the literature and this study, Ti allergy typically occurred in implant patients suffering from postoperative complaints. We identified erythema, dermatitis (overlying the implant or elsewhere), and local swelling. Patients also reported pruritus, a burning sensation, and pain. These results are in line with those of previous studies. However, the relationship between implant‐related complaints and allergy remains much debated. Determining the relevance of a positive test reaction in these patients is a challenge. Although we describe relevance in 61.5%, it was difficult to determine whether a positive reaction was putatively responsible for the clinical complaints. Given the multifactorial background of the above‐mentioned complaints, other factors, such as aseptic loosening, osteolysis and infection, may also play a significant role.^27^ Moreover, possible sensitization to other components within the implant makes it complex to determine whether Ti is the primary cause, is an aggravating factor, or is not involved at all in the pathogenesis of the clinical complaints. It was sometimes difficult, and often impossible, to retrospectively determine the relative influences of all these different factors; hence the high amount of partial relevance in our study. Sufficient information on previous diagnostic outcomes and implanted materials is therefore crucial. Fortunately, the composition of dental implants and reconstructive materials can be determined by taking microsamples.^28^ This method also detects possible trace metals that are not listed by the manufacturer or registered by the clinician, but may be involved in the clinical manifestation. In addition, a study by Bernard et al showed the presence of many impurities in commercialized Ti patch test samples.^23^ Even though there is no proof that the level of impurities in the patch test Ti salts is sufficient to cause elicitation, it highlights the importance of testing all implanted materials and investigating the existence of allergenic exposures.

Our study had several limitations. It was a retrospective study, so it was difficult to assign relevance, designate a control group, and rule out the possibility of referral bias occurring. In addition, not all of the patients were screened with the same salts, as the Ti series has expanded over the last 10 years. However, our study is the first to test a panel of Ti salts on a large patch test population. Future prospective studies could avoid these limitations, and further assess the accuracy of Ti patch test salts.

In conclusion, the frequency of Ti sensitivity in our patch test population was 5.7%. The alternative Ti patch test salts evaluated in this study enhance the diagnostic work‐up, as they are possibly superior to TiO_2_ as test salts for patch testing. However, a single Ti salt cannot be used as a patch test preparation, as patient‐specific responses occur to different salts, their accuracy in diagnosing Ti sensitization is mostly unknown, and determining the relevance of a positive result is still challenging. This illustrates the problems that clinicians face in evaluating Ti allergy, indicating that large‐scale prospective studies are necessary to develop new patch test salts and improve alternative diagnostic tools such as the LTT. It remains important to diagnose on a case‐by‐case basis, duly taking the medical history and the clinical picture into account.

### Conflict of interest

The authors declare no potential conflict of interests.

## Supporting information


**Table S1** Titanium test salt combinations.
