# Supplementary material for: A retrospective study on titanium sensitivity: Patch test materials and manifestations
Source: Contact Dermatitis. 2018 May 24;79(2):85–90. doi: 10.1111/cod.13010 (PMC6099462; doi:10.1111/cod.13010)
Supplement: Supplementary file 1 — Table S1 Titanium test salt combinations. [file COD-79-85-s001.docx]

# Online Supplementary Tables

**Supplementary Table 1.** Titanium test salt combinations

|  |  | **N.** |
| --- | --- | --- |
| **Tested for Ti sensitization** |  | **458** |
| **Tested for Ti sensitization with:** |  |  |
| Only Ti dioxide |  | 183 |
| Only Ti (IV) oxalate hydrate |  | 2 |
| Only Ti (IV) isopropoxide |  | 20 |
| Ti dioxide & Ti (IV) oxalate hydrate |  | 1 |
| Ti dioxide & Ti (IV) isopropoxide |  | 38 |
| Ti (IV) oxalate hydrate & Ti (IV) isopropoxide |  | 107 |
| Ti dioxide & Ti (IV) oxalate hydrate & Ti (IV) isopropoxide |  | 62 |
| Ti dioxide & Ti (IV) isopropoxide & Ti lactate & Ti citrate |  | 1 |
| Tested with all Ti salts |  | 44 |
